# A balancing act: investigations on the impact of altered signal sensitivity in bacterial quorum sensing

**DOI:** 10.1128/jb.00249-23

**Published:** 2023-11-27

**Authors:** Samantha Wellington Miranda, E. Peter Greenberg

**Affiliations:** 1 Department of Microbiology, University of Washington, Seattle, Washington, USA; University of California San Francisco, San Francisco, California, USA

**Keywords:** cell-cell signaling, *Pseudomonas aeruginosa*, quorum sensing, signal sensitivity, interspecies interactions

## Abstract

Many bacterial species communicate and cooperate with kin using a form of cell-cell signaling called quorum sensing. Sensitivity and selectivity are fundamental properties of quorum sensing, and of cell signaling more generally, as they dictate the ability of cells to accurately detect and interpret signals. In this study, we investigate the impact of signal sensitivity on gene regulation and on bacterial fitness using two variants of the LasR quorum sensing receptor in the opportunistic pathogen *Pseudomonas aeruginosa*. Our studies reinforce that altering LasR signal-binding residues tends to result in decreased signal selectivity. We also find that LasR hyper-sensitivity results in earlier and stronger expression of LasR-regulated genes, whereas hypo-sensitivity results in delayed transcription from both LasR- and RhlR-regulated promoters. Furthermore, strains expressing either the hyper- or hypo-sensitive LasR polypeptide exhibit reduced production of pyocyanin, a key antimicrobial that impacts intra- and interspecies competition as well as pathogenicity. These changes to gene expression underscore the complex regulatory network by which *P. aeruginosa* controls group behaviors. Ultimately, altered signal sensitivity results in a fitness defect for LasR hyper-sensitive cells during kin competition against wild-type *P. aeruginosa* and for hypo-sensitive cells during interspecies competition against the opportunistic pathogen *Burkholderia multivorans*. Our findings highlight the delicate balance between group behaviors regulated by quorum sensing and bacterial fitness and contribute to a better understanding of the evolutionary pressures that may tune sensitivity in cell-cell signaling.

**IMPORTANCE:**

Quorum sensing (QS) is a widespread form of cell-cell signaling that regulates group behaviors important for competition and cooperation within bacterial communities. The QS systems from different bacterial species have diverse properties, but the functional consequences of this diversity are largely unknown. Taking advantage of hyper- and hypo-sensitive QS receptor variants in the opportunistic pathogen *Pseudomonas aeruginosa*, we examine the costs and benefits of altered signal sensitivity. We find that the sensitivity of a model QS receptor, LasR, impacts the timing and level of quorum gene expression, and fitness during intra- and interspecies competition. These findings suggest competition with kin and with other bacterial species work together to tune signal sensitivity.

## INTRODUCTION

Cell-cell signaling plays a crucial role in bacterial communities, enabling coordinated group behaviors key to both symbiosis and pathogenesis (1–3). Signal selectivity and sensitivity are fundamental parameters of signaling that dictate under which conditions a response will be generated. Much attention has been given to the molecular mechanisms and evolution of signaling selectivity (4–9). Here, we turn our attention to the evolution and function of signaling sensitivity, focusing on bacterial quorum sensing (QS) as a model. QS is a widespread form of bacterial communication that links gene expression to cell density (3). QS systems often regulate group behaviors such as antibiotic and toxin synthesis, biofilm formation, and exoenzyme production. In many Proteobacteria, QS is mediated by small-molecule acyl-homoserine lactone (AHL) signals. Generally, in these systems, a LuxI-type signal synthase produces an AHL signal, and a paired cytosolic LuxR-type receptor regulates gene expression in response to signal binding. The QS receptors from different bacterial species display a wide range of signal sensitivity, spanning from picomolar to micromolar, and a wide range of selectivity, from specific to highly promiscuous (5). The optimal sensitivity of a given receptor is likely influenced by numerous factors, including the functions and metabolic cost of the processes regulated by the receptor, the amount of signal produced by the paired signal synthase, and the physical properties of the signal and of the environment(s) the bacterial species inhabits.

**Fig 1:**
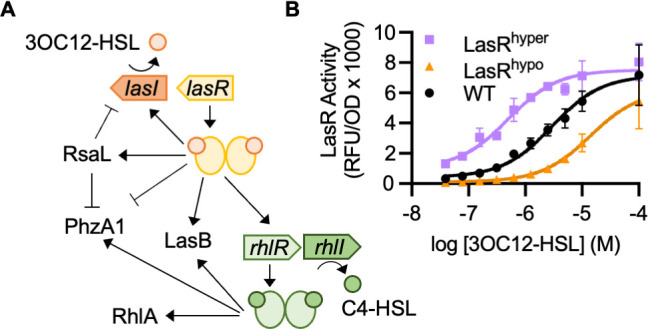
The *P. aeruginosa* AHL QS network and influence of *lasR* mutations on signal sensitivity. (**A**) LasR activates the transcription of several genes including *lasI, rsaL,* and *lasB*. LasR also, both directly and indirectly, inhibits transcription of the phenazine biosynthesis gene *phzA1*. In strain PAO1, LasR positively regulates the Rhl QS system, which itself activates several genes including *lasB, rhlA,* and *phzA1*. (**B**) LasR activity in response to 3OC12-HSL measured in *P. aeruginosa* ∆*lasI* ∆*rhlI* (PAO-SC4) with wild-type (WT) or variant LasR. Activity was measured as relative fluorescence units (RFU) normalized to optical density at 600 nm (OD) using the pBBR-P*_rsaL_-gfp* transcriptional reporter. Data are the mean and standard deviation of two biological replicates (i.e., two experimental cultures inoculated from the same initial culture and grown in parallel) and are representative of three independent experiments (i.e., experiments conducted on separate days).

The opportunistic pathogen *Pseudomonas aeruginosa* is a well-studied model organism for investigating the molecular biology and evolution of QS (3). *P. aeruginosa* has two complete AHL QS circuits with both distinct and overlapping transcriptional regulons (Fig. 1A). LasI-LasR uses the signal *N*-(3-oxododecanoyl)-L-homoserine lactone (3OC12-HSL), and RhlI-RhlR uses the signal *N*-butyryl-L-homoserine lactone (C4-HSL). In the laboratory strain PAO1, LasR positively regulates the Rhl system and is required for RhlR activity under standard culture conditions. Together, these QS systems regulate over 200 genes, several of which encode key virulence factors including the secreted protease LasB (elastase), the redox-active antimicrobial pyocyanin, and biosurfactant rhamnolipids (10–12). The Las and Rhl QS systems are embedded in a complex regulatory network that contains both positive and negative feedback loops. For instance, LasR activates the transcription of *lasI*, the gene encoding the 3OC12-HSL signal synthase, resulting in a positive feedback loop. LasR also activates the transcription of *rsaL*, which encodes a negative regulator of *lasI*, resulting in a negative feedback loop.

We recently conducted a covariation analysis of AHL QS systems to identify residues in LasR that coevolve with residues in LasI (6). We had hypothesized that these amino acids would be important for signal selectivity, and indeed that is the case. We also observed that substitutions in the covarying residues alter signal sensitivity, indicating that perhaps sensitivity and selectivity are strongly linked or that LuxI and LuxR homologs coevolve for both sensitivity and selectivity. Of particular interest were two variants of LasR that are more sensitive to 3OC12-HSL than wild-type (WT), indicating that LasR has not evolved for maximal signal sensitivity (6). In our present study, we sought to uncover why this would be and to identify selective pressures that tune QS signal sensitivity. In *P. aeruginosa,* the fitness benefits from QS are greater at higher cell densities (13), and receptor sensitivity is one factor that could determine when during cell growth the QS regulon is activated. Furthermore, both the timing and magnitude of QS-dependent gene expression are tightly controlled in *P. aeruginosa* and in many other bacterial species by negative regulatory elements (14). Examples of these inhibitory regulators include RsaL, QscR, QslA, and QteE in *P. aeruginosa* and TrlR and TraM in *Agrobacterium tumefaciens* (15–17). The frequent presence of negative regulators of QS suggests that receptor hyper-sensitivity would be a detriment to bacterial fitness, but this hypothesis has not been explicitly tested. Conversely, receptor hypo-sensitivity may impair growth in certain environments and may affect the ability to produce molecules required to compete with kin cells or with other bacterial species.

In this study, we use a hyper- and a hypo-sensitive LasR variant to examine the consequences of altered QS sensitivity for *P. aeruginosa*. The LasR^hyper^ polypeptide has the amino acid substitution A127L and is 6-fold more sensitive to 3OC12-HSL than WT, while the LasR^hypo^ polypeptide has the substitution L125F and is 10-fold less sensitive than WT (Fig. 1B). We show that these LasR variants, which shift signal sensitivity, can alter the timing and level of QS gene activation as well as the signal selectivity of LasR in *P. aeruginosa* cells. Ultimately, these changes to gene regulation result in decreased fitness in intra- and interspecies competition. These findings suggest that signal sensitivity is finely tuned to balance costly group behaviors with bacterial fitness in the context of polymicrobial communities where bacteria compete with both kin cells and other bacterial species.

## RESULTS

### LasR variants have reduced selectivity

**Fig 2:**
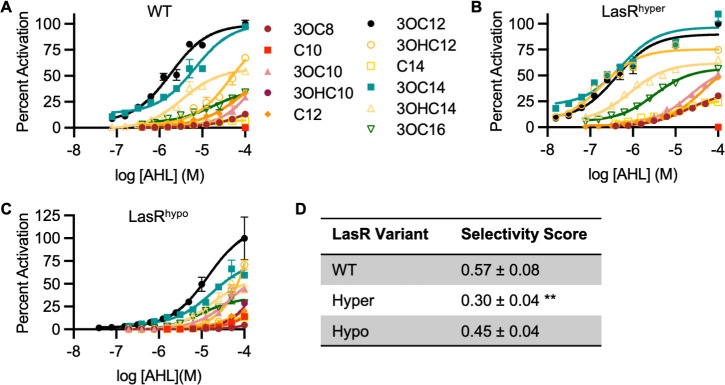
LasR variants are less selective than WT. LasR activity in response to a panel of AHL signals (Fig. S1) was measured using pBBR-P_rsaL_-*gfp* in the synthase-null strain PAO-SC4. GFP fluorescence was normalized to cell density (optical density at 600 nm), and values were then normalized to the maximal activity of LasR stimulated by 3OC12-HSL to yield “percent activation.” Activity was measured in (**A**) PAO-SC4-WT, (**B**) -LasR^hyper^, or (**C**) -LasR^hypo^. Data points are the mean and standard deviation of two biological replicates and are representative of three independent experiments. (**D**) Selectivity scores for each LasR variant were calculated from dose-response experiments (representative results shown in panels A, B, and C). Values are the mean and standard deviation of the scores from three independent experiments. ** indicates significantly different from WT; *P* < 0.002, ANOVA).

One consequence of increased receptor sensitivity may be decreased selectivity, i.e., the receptor may also have increased sensitivity to non-self-signals (5–7). We previously found that *P. aeruginosa* expressing LasR^hyper^ has increased sensitivity to several non-self-signals (6), but we did not quantitatively assess whether its selectivity is greater or less than that of WT. Likewise, we previously found that cells expressing LasR^hypo^ are more responsive to 3OC10-HSL than WT, but selectivity toward other signals was not studied. To assess the selectivity of our variants, we measured LasR activity in response to the panel of AHL signals shown in Fig. S1 by using AHL synthase-null (*lasI^–^ rhlI^–^*) *P. aeruginosa* (PAO-SC4; see Tables S1 and S2 for descriptions of bacterial strains and plasmids used in this study). Because signal sensitivity and selectivity are significantly impacted by changes to receptor expression level, e.g., due to expression from a non-native promoter or from a plasmid (5), we used strains with unmarked mutations in the native *lasR* locus that code for LasR^hyper^ or LasR^hypo^. We found that in addition to responding more sensitively to 3OC12-HSL than PAO-SC4-WT, PAO-SC4-LasR^hyper^ responds with equal sensitivity to 3OC12-HSL and the non-self-signal *N-*3-(oxotetradecanoyl)-L-homoserine lactone (3OC14-HSL) (Fig. 2A and B). The half maximal effective concentration (EC_50_) and the relative level of activation by each signal for each LasR variant are listed in Table S3. PAO-SC4-LasR^hyper^ also has increased sensitivity to several other signals in the panel, responding with a lower EC_50_ than PAO-SC4-WT and with a higher level of maximal activity relative to the cognate signal 3OC12-HSL. Although PAO-SC4-LasR^hypo^ loses sensitivity to 3OC12-HSL, it retains moderate sensitivity to 3OC14-HSL as well as to several other signals (Fig. 2C). We calculated a signal selectivity score for each LasR variant using a previously developed formula that takes into account both sensitivity and maximal activity stimulated by each signal in the panel by comparing the area under the curve (AUC) for activity stimulated by 3OC12-HSL to the AUC for all other signals (5). PAO-SC4-LasR^hyper^ has a significantly lower score than PAO-SC4-WT, indicating that LasR^hyper^ is less selective than WT LasR (Fig. 2D). PAO-SC4-LasR^hypo^ also has a lower selectivity score than WT, but this difference is not statistically significant. To better understand this result, we measured the selectivity of an additional hypo-sensitive variant, PAO-SC4-LasR^R61L^, which is significantly impaired for 3OC12-HSL sensitivity (EC_50_ >20 µM) (6). This variant is also less selective than WT (selectivity score = 0.20 ± 0.03, *P* < 0.001, ANOVA) (Fig. S2). These results suggest that LasR has evolved for selectivity toward 3OC12-HSL, and mutations that affect LasR ligand-binding residues are likely to decrease this selectivity.

### LasR variants display altered quorum gene expression

To determine the impact of LasR sensitivity on gene regulation, we measured the transcriptional activity from the promoters of four quorum-regulated genes: (i) *rsaL,* which is activated by LasR; (ii) *lasB,* which is activated by both LasR and RhlR; (iii) *rhlA*, a gene required for rhamnolipid biosynthesis, which is activated by RhlR; and (iv) *phzA1*, a gene required for the production of pyocyanin and related phenazines, which is positively regulated by RhlR but is both directly and indirectly negatively regulated by LasR (Fig. 1A) (15, 18, 19). Because LasR positively regulates the Rhl system, altered LasR sensitivity may impact RhlR activity, but RhlR activity is also dependent upon several other factors (20–23). Furthermore, given the multiple overlapping regulatory networks acting on these genes, the timing of quorum gene expression may be robust against perturbations in a single regulator. It has previously been shown that exogenous addition of AHL or early expression of LasR is insufficient to significantly advance the expression of most quorum-regulated genes in *P. aeruginosa* (11). To measure transcriptional activity, we used fluorescent reporters in which the quorum-regulated promoters control *gfp* expression in *P. aeruginosa* with chromosomal *lasR* mutations. All reporter strains grew equivalently in our experimental conditions and had similar background fluorescence measured from a promoterless control (Fig. S3A through D).

**Fig 3:**
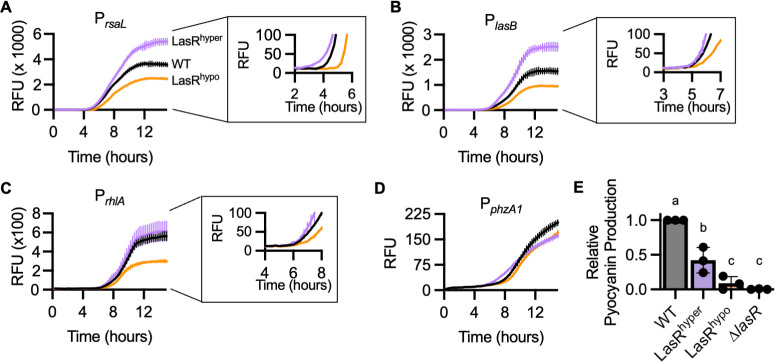
Receptor sensitivity affects transcriptional activation. (**A**) Transcriptional activity from the *rsaL* promoter measured as GFP fluorescence [relative fluorescence units (RFU)] from pBBR-P*_rsaL_-gfp* in PAO1-WT (black), -LasR^hyper^ (purple), or -LasR^hypo^ (orange). Inset shows same data, with axes modified to visualize early time points. (**B**) Transcriptional activity from the *lasB* promoter measured as GFP fluorescence (RFU) from pBBR-P*_lasB_-gfp* in PAO1-WT (black), -LasR^hyper^ (purple), or -LasR^hypo^ (orange). Inset shows same data, with axes modified to visualize early time points. (**C**) Transcriptional activity from the *rhlA* promoter, measured as GFP fluorescence (RFU) from pBBR-P*_rhlA_-gfp* in PAO1-WT (black), -LasR^hyper^ (purple), or -LasR^hypo^ (orange). Inset shows same data, with axes modified to visualize early time points. (**D**) Transcriptional activation from the *phzA1* promoter, measured as GFP fluorescence (RFU) using pBBR-P*_phzA1_-gfp* in PAO1-WT (black), -LasR^hyper^ (purple), or -LasR^hypo^ (orange). In A–D, lines show the mean and standard deviation of three biological replicates and are representative of three to five independent experiments. (**E**) Relative pyocyanin concentration in cultures of PAO1-WT, -LasR^hyper^, -LasR^hypo^, or -∆*lasR* grown for 20–22 hours in pyocyanin production medium, normalized to WT. Each data point is the mean of three biological replicates within an independent experiment. Lines show the mean and standard deviation of the data points. Data annotated by different letters are significantly different (*P* < 0.02, ANOVA).

Compared to PAO1-WT, PAO1-LasR^hyper^ activated transcription from the *rsaL* promoter earlier during cell growth and to a higher overall level, whereas PAO1-LasR^hypo^ had delayed and lower activity at this promoter (Fig. 3A; Fig. S3E). This demonstrates that 3OC12-HSL sensitivity can affect the timing and magnitude of LasR activity. PAO1-LasR^hyper^ also activated P*_lasB_* to a higher degree than PAO1-WT, but the timing of P*_lasB_* activation was similar between the two strains (Fig. 3B; Fig. S3F), suggesting that other regulators, such as RhlR, may limit the timing of *lasB* transcription. Although LasR positively regulates the Rhl system, PAO1-LasR^hyper^ and -WT displayed indistinguishable P*_rhlA_* activity (Fig. 3C; Fig. S3G), indicating that factors other than LasR limit Rhl activity in our culture conditions. PAO1-LasR^hypo^ displayed delayed and reduced activity at both the *lasB* and *rhlA* promoters, suggesting that in the hypo-sensitive mutant, LasR becomes a limiting regulator for these two genes (Fig. 3B and C; Fig. S3F and G). Finally, both PAO1-LasR^hyper^ and -LasR^hypo^ had lower transcriptional activity at the *phzA1* promoter than PAO1-WT (Fig. 3D, Fig. S3H). Because this pattern of activity diverged from that of the other promoters, we directly measured pyocyanin production in WT and LasR-variant *P. aeruginosa*. Consistent with the transcriptional reporter data, both PAO1-LasR^hyper^ and -LasR^hypo^ produced significantly less pyocyanin than WT (Fig. 3E). In PAO1-LasR^hyper^, pyocyanin production was less than half of WT levels and could result from increased expression of the transcriptional repressor RsaL (15). In cultures of PAO1-LasR^hypo^, pyocyanin production was about 9% of WT levels and not significantly different from PAO1-∆*lasR*. This could be the result of lower RhlR activity (24).

### LasR sensitivity alters fitness during kin competition

**Fig 4:**
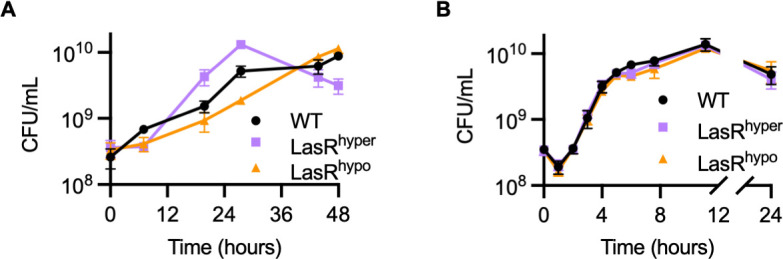
Receptor sensitivity affects growth in a QS-requiring medium. Growth of PAO1-WT, -LasR^hyper^, or -LasR^hypo^ in (**A**) casein broth or (**B**) casamino acids broth measured as CFU per milliliter. Data are the mean and standard deviation of three biological replicates and are representative of three independent experiments.

To determine whether altered LasR sensitivity affects *P. aeruginosa* fitness, we used an *in vitro* growth model in which the protein casein serves as the sole carbon and energy source. *P. aeruginosa* requires quorum-regulated proteases such as LasB to digest casein as a nutrient source. Strains lacking functional LasR do not express *lasB* and are unable to grow in this medium (25, 26). Consistent with altered activation of the *lasB* promoter, PAO1-LasR^hyper^ grew more quickly in casein broth than PAO1-WT, while PAO1-LasR^hypo^ grew more slowly (Fig. 4A). The accelerated growth of PAO1-LasR^hyper^ was followed by an approximately 4-fold reduction in colony-forming units (CFU) over the subsequent 20 hours of incubation. Growth of the LasR variants was indistinguishable from WT in our control medium casamino acids broth (Fig. 4B). Casamino acids are derived from casein by acid hydrolysis, and growth of *P. aeruginosa* on casamino acids broth does not require QS.

**Fig 5:**
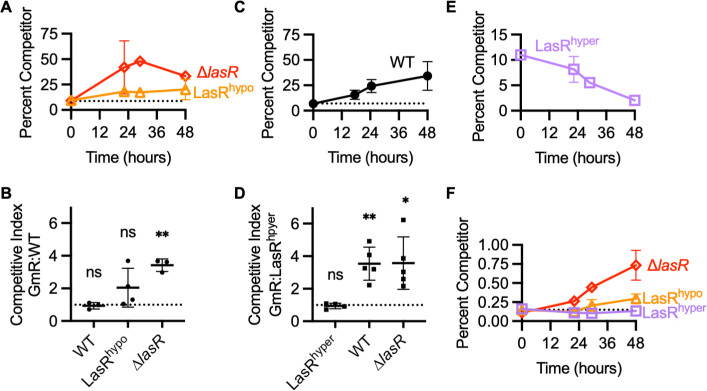
Receptor sensitivity affects fitness in intraspecies competition. (**A**) Frequency of PAO1-LasR^hypo^-GmR (triangles) or of PAO1-∆*lasR*-GmR (diamonds) in casein broth coculture with PAO1-WT. Data are the mean and standard deviation of three biological replicates and are representative of three to four independent experiments. (**B**) Competitive index of the indicated GmR competitor after 48 hours of coculture with PAO1-WT in casein broth. Cultures were inoculated at a ratio of 9:1 WT:GmR competitor. Each data point shows the mean of an independent experiment consisting of three biological replicates. Lines show the mean and standard deviation of the data points. The dashed line marks a competitive index of 1, which indicates the two cocultured strains are equally fit. (**C**) Frequency of PAO1-WT-GmR in casein broth coculture with PAO1-LasR^hyper^. Data are the mean and standard deviation of three biological replicates and are representative of five independent experiments. (**D**) Competitive index of the indicated GmR competitor after 48 hours of coculture with PAO1-LasR^hyper^ in casein broth. Cultures were inoculated at a ratio of 9:1 LasR^hyper^:GmR competitor. Each data point shows the mean of an independent experiment consisting of three biological replicates. Lines show the mean and standard deviation of the data points. In B and D, * indicates *P* < 0.03, ** indicates *P* < 0.01, ns is not significant (one-sample *t*-test, hypothetical mean = 1.0). (**E**) Frequency of PAO1-LasR^hyper^-GmR in casein broth coculture with PAO1-WT. Cultures were inoculated at a ratio of 9:1 WT:GmR competitor. Data are the mean and standard deviation of three biological replicates and are representative of three independent experiments. (**F**) Frequency of PAO1-∆*lasR*-GmR (diamonds), PAO1-LasR^hypo^-GmR (triangles), or PAO1-LasR^hyper^-GmR (squares) in casein broth coculture with PAO1-WT. The inoculum contained 0.1% GmR competitor. Data are the mean and standard deviation of three biological replicates and are representative of three to four independent experiments.

In casein broth coculture, the secreted proteases produced by quorum-competent “cooperator” cells can be used by “cheater” cells that have a loss of function in LasR (∆LasR) and do not produce protease. Because ∆LasR cheaters gain a benefit without incurring the cost of an active QS response, these cells have a significant fitness advantage over WT in casein broth coculture (25, 26). When *P. aeruginosa* PAO1 is passaged in casein broth, ∆LasR cheaters emerge and reach a stable equilibrium of about 30%–40% (25–27). We reasoned that similarly, strains that have lower LasR activity than a competitor could gain a fitness advantage by exploiting protease produced by the other strain in casein broth coculture. To test this hypothesis, we competed *P. aeruginosa* with various *lasR* mutations against either PAO1-WT or -LasR^hyper^. The competitor strains were marked with gentamicin resistance (GmR), and we started our cultures with a frequency of 10% GmR competitor because this would allow reliable detection of increases or decreases in competitor frequency. In addition, this frequency has been used in past studies of the competition between PAO1-WT and PAO1-∆*lasR*, making this condition a control for which we know the expected outcome (28). Although PAO1-LasR^hypo^ has lower QS activity than WT, it does not have a clear fitness advantage against PAO1-WT in casein broth (Fig. 5A; Fig. S4A). The outcome of this competition was variable across biological replicates and across four independent experiments, with one to three of three biological replicates showing a fitness advantage for PAO1-LasR^hypo^-GmR (Fig. S4B). This variability resulted in a low averaged competitive index for PAO1-LasR^hypo^-GmR competed against PAO1-WT (Fig. 5B). This contrasts with PAO1-∆*lasR*-GmR which consistently enriched from a starting frequency of 10% to a final frequency of 30%–40% in coculture with PAO1-WT in our experiments as well as in other published studies (Fig. 5A and B; Fig. S4C and D) (25, 26, 28). As a control, we also competed PAO1-WT-GmR against the unmarked PAO1-WT parent and found that the strains are equally fit in casein broth coculture (Fig. 5B; Fig. S4E).

Because PAO1-LasR^hyper^ activates quorum-regulated genes more strongly than PAO1-WT, we reasoned that PAO1-WT may have a fitness advantage against PAO1-LasR^hyper^ in coculture. Indeed, despite growing more slowly in monoculture, in casein broth coculture with PAO1-LasR^hyper^, PAO1-WT-GmR enriched from an initial frequency of 10% to a final frequency of approximately 30% (Fig. 5C; Fig. S5A and B). This fitness advantage is similar to that of PAO1-∆*lasR*-GmR competed against PAO1-WT (Fig. 5D) and is apparent before cell death begins for PAO1-LasR^hyper^. PAO1-∆*lasR*-GmR also had a strong advantage against PAO1-LasR^hyper^, increasing from 10% to 55% of the coculture after 48 hours, on average (Fig. S5C and D).

Quorum-regulated genes are expressed at a low level in casamino acids broth compared to casein broth, and accordingly, PAO1-∆*lasR* does not have a fitness advantage against PAO1-WT in casamino acids broth (25). Consistent with previous studies, PAO1-∆*lasR*-GmR did not have a significant advantage against PAO1-WT in casamino acids broth coculture in our present study (Fig. S6A). PAO1-LasR^hypo^-GmR also displayed equal fitness to PAO1-WT in casamino acids broth coculture. Both PAO1-WT-GmR and -∆*lasR*-GmR, however, showed a slight fitness advantage against PAO1-LasR^hyper^ in casamino acids broth coculture (Fig. S6B and C). This advantage is statistically significant for PAO1-∆*lasR*-GmR but not PAO1-WT-GmR and may reflect higher QS activity in PAO1-LasR^hyper^ grown in casamino acids broth compared to PAO1-WT (Fig. S6D and E).

To further investigate the fitness advantage of PAO1-WT over PAO1-LasR^hyper^ in casein broth, we cocultured PAO1-LasR^hyper^-GmR and PAO1-WT with a starting frequency of 10% GmR competitor. Under these conditions, PAO1-LasR^hyper^-GmR decreased in relative abundance to a final frequency of 4%, on average (Fig. 5E; Fig. S7A). The advantage for PAO1-WT was evident across additional inoculum ratios of PAO1-WT to PAO1-LasR^hyper^. When included as 1% of the inoculum, PAO1-WT-GmR increased in relative abundance, whereas when PAO1-LasR^hyper^-GmR comprised 1% of the inoculum, its frequency decreased (Fig. S7B). These outcomes were reflected in competitive indices >1 for PAO1-WT-GmR when competed against PAO1-LasR^hyper^ and <1 for PAO1-LasR^hyper^-GmR when competed against PAO1-WT, consistent with the fitness advantage for PAO1-WT in each of the competitions (Fig. S7C).

Finally, if LasR variants were to evolve in a population of WT *P. aeruginosa*, they would initially be at a very low frequency of the total population. To infer the competitive fitness of our LasR variants in such a setting, without incurring other evolutionary changes, we assessed the ability of LasR variant strains to invade PAO1-WT in casein broth from a rare initial frequency. When included at 0.1% of the inoculum, PAO1-∆*lasR*-GmR effectively invaded PAO1-WT, enriching 7-fold on average. PAO1-LasR^hypo^-GmR and PAO1-LasR^hyper^-GmR had no such advantage. When inoculated at an initial frequency of 0.1%, both variants remained near the starting frequency (Fig. 5F; Fig. S8A). At a 1% initial frequency, PAO1-∆*lasR*-GmR had a significant fitness advantage against PAO1-WT (competitive index = 8.7), whereas PAO1-LasR^hypo^-GmR had only a slight advantage (competitive index = 1.9) (Fig. S8B and C). As stated above, when inoculated at 1% of a coculture with PAO1-WT, PAO1-LasR^hyper^-GmR decreased in abundance (competitive index = 0.4) (Fig. S7B and C).

### LasR hypo-sensitivity impairs fitness during interspecies competition

**Fig 6:**
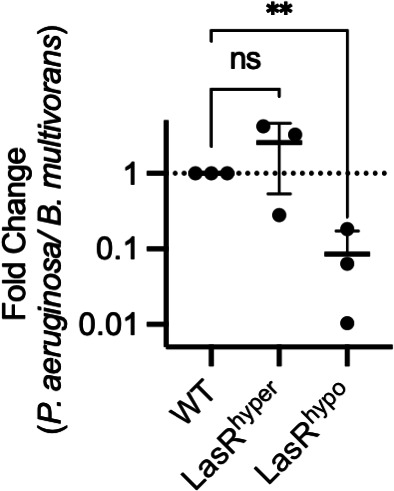
LasR sensitivity impacts interspecies competition. PAO1-WT, -LasR^hyper^, or -LasR^hypo^ was competed against *B. multivorans* in buffered lysogeny broth (LB supplemented with 3-(*N*-morpholino)-propanesulfonic acid (MOPS)). The final ratio of CFU/mL of *P. aeruginosa* to *B. multivorans* after 24 hours of coculture was normalized to the ratio of PAO1-WT to *B. multivorans*. Each point represents the mean of an independent experiment consisting of three biological replicates. Lines show the mean and standard deviation of data points. ** indicates *P* < 0.01, ns is not significant (ANOVA).

Within the *P. aeruginosa* QS regulon are genes coding for multiple antimicrobial molecules capable of killing both bacterial and eukaryotic cells. Phenazines, such as pyocyanin, play a key role in these competitive interactions (29–31). Given the reduced production of pyocyanin by both PAO1-LasR^hyper^ and -LasR^hypo^, we hypothesized the variants would have a defect in interspecies competition relative to WT. To test this, we used an established coculture model of *P. aeruginosa* and the opportunistic pathogen *Burkholderia multivorans. B. multivorans* is a member of the *Burkholderia cepacia* complex and can coexist with *P. aeruginosa* in soil and in lung infections of individuals with the genetic disease cystic fibrosis (CF). In coculture, *P. aeruginosa* kills *B. multivorans* through the production of quorum-regulated phenazines, rhamnolipids, and hydrogen cyanide (31). PAO1-LasR^hypo^ has a significant defect in this model, resulting in 10-fold less killing of *B. multivorans* than PAO1-WT (Fig. 6). PAO1-LasR^hyper^, on the other hand, kills *B. multivorans* as well as PAO1-WT in this model. This could reflect equal activation of rhamnolipid genes (*rhlA*) in PAO1-WT and -LasR^hyper^ and potentially of other RhlR-regulated products such as hydrogen cyanide.

## DISCUSSION

In *P. aeruginosa*, and in other bacterial species, QS is embedded within a complex regulatory network. The timing and level of quorum gene activation are subject to multiple levels of regulation, including positive and negative feedback loops. This architecture perhaps evolved to balance the high metabolic cost of quorum-regulated processes with competition from kin cells. Indeed, kin competition has been demonstrated to be an evolutionary pressure on QS signal selectivity in *Bacillus subtilis* (32). Here, we investigate the consequences of altering QS signal sensitivity by using the model system LasI-LasR from *P. aeruginosa*. LasR^hyper^ is 6-fold more sensitive to the LasR cognate signal 3OC12-HSL, and LasR^hypo^ is 10-fold less sensitive than WT. Increases to signal sensitivity often result in reduced selectivity (6, 7). LasR^hyper^ is not only more sensitive to 3OC12-HSL, it is also more sensitive to several non-self-signals resulting in a more promiscuous receptor. In addition, LasR^hypo^ trends toward more promiscuity than WT, and an additional hypo-sensitive LasR variant is significantly more promiscuous than WT. These results suggest that LasR has evolved for selectivity toward its native signal, and changes to the ligand-binding site disrupt this selectivity. Even single amino acid substitutions significantly decrease selectivity and increase the potential for cross talk between *P. aeruginosa* and other AHL-producing bacterial species. This may be a common feature of selectivity in signaling. A recent study on bacterial two-component signaling found that these systems possess only “marginal specificity,” with minor changes to amino acid sequence resulting in substantial cross talk (9).

We hypothesized that changes to LasR sensitivity would impact both the timing and level of quorum gene activation. Using transcriptional reporters, we found that LasR hyper-sensitivity results in earlier and higher activation of the LasR-regulated *rsaL* promoter and higher activation of the *lasB* promoter. LasR hypo-sensitivity results in the opposite: delayed and lower activation of the promoters for both *rsaL* and *lasB*. Although LasR activates the Rhl system, increased LasR activity in PAO1-LasR^hyper^ is not sufficient to result in earlier or stronger expression from the RhlR-regulated *rhlA* promoter. Conversely, LasR hypo-sensitivity delays and reduces expression from the *rhlA* promoter, suggesting that, in this strain, LasR has become a limiting regulator of RhlR activity. Furthermore, both LasR hyper- and hypo-sensitivity resulted in decreased production of the antimicrobial molecule pyocyanin. These results contrast with strains lacking the anti-activators QscR, QslA, and/or QteE, which have a hyper-active LasR phenotype but produce significantly more pyocyanin than WT *P. aeruginosa* (17, 33). This difference could potentially be a result of increased RhlR activity in the anti-activator mutants but not in PAO1-LasR^hyper^. Our studies highlight the complexity of the regulatory web in which LasR is embedded and suggest that pyocyanin production depends in part on the balance between LasR and RhlR activity.

Ultimately, we assessed whether the influence of LasR^hyper^ or LasR^hypo^ on gene regulation affects the fitness of *P. aeruginosa*. Quorum-regulated exoproducts are required to access nutrients for growth in certain environments as well as to compete with neighboring bacterial species and invade host organisms. For example, in culture media containing a protein (e.g., casein) as the sole carbon source, QS-regulated proteases such as LasB are required to digest the protein and enable cell growth. Although strains lacking functional LasR cannot grow on proteins as a nutrient source, when cocultured with WT *P. aeruginosa*, these strains exploit protease produced by WT and have a fitness advantage against the WT cells (25, 26, 34). In casein broth monoculture, LasR hypo-sensitivity results in slower growth. But, unlike PAO1-∆*lasR*, PAO1-LasR^hypo^ does not have a strong fitness advantage against WT in casein broth coculture and does not effectively invade PAO1-WT from a relatively rare starting frequency of 0.1% or 1%. Furthermore, PAO1-LasR^hypo^ has a defect in an interspecies competition against the pathogen *B. multivorans*, consistent with underproduction of pyocyanin and rhamnolipids by the hypo-sensitive strain. Given that multiple antimicrobial molecules are regulated by QS in *P. aeruginosa*, it is plausible that interspecies competition imparts a significant pressure to retain QS function. In support of this hypothesis, it has been shown that compared to monoculture, *P. aeruginosa* is less likely to acquire mutations in *lasR* when evolved in coculture with a model polymicrobial community containing typical CF pathogens, including *Burkholderia cenocepacia* (another member of the *Burkholderia cepacia* complex) (35). Conversely, despite growing faster in casein broth monoculture, PAO1-LasR^hyper^ is less fit than PAO1-WT in casein broth coculture across multiple starting ratios. This result suggests that competition with kin selects against increased signal sensitivity.

In addition to providing insight into the consequences of and selective pressures on signaling sensitivity, our findings also have implications for synthetic biology and directed evolution. AHL QS is of particular interest in synthetic biology for engineering coordinated behaviors in bacterial populations and stabilizing bacterial consortia (36). However, cross talk between well-studied QS systems limits their potential applications. As such, researchers desire to design or evolve QS systems to respond to new signals and to limit cross talk (37). The observation that even a relatively small increase to signal sensitivity can result in a fitness disadvantage against kin cells suggests that selective conditions used in experimental evolution should employ a private good [e.g., growth in adenosine broth for LasR (26) or regulation of cell-intrinsic antibiotic resistance] rather than a public good [e.g., growth in casein broth for LasR or regulation of antibiotic resistance that could be shared by a population of cells, such as β-lactamases (38, 39)]. Furthermore, given the propensity for single amino acid substitutions to increase receptor promiscuity, negative selection against unwanted responses should be included when evolving or designing new QS systems.

Collectively, our studies highlight the complex regulatory network by which *P. aeruginosa* coordinates group behaviors and controls the timing and level of metabolically costly products. Our findings suggest evolutionary pressures, including kin competition, interspecies competition, and nutrient acquisition, work together to fine-tune sensitivity in cell-cell signaling. Of course, one could argue that our work on the fitness of hyper- and hypo-sensitive LasR variants is limited to laboratory-generated alleles and laboratory conditions and leaves in question what might be selective pressures that drive the selectivity and sensitivity of QS signal receptors like LasR in nature. Furthermore, although we speculate that our findings would apply to other variants with altered signal sensitivity, we have only investigated one variant of each type, and it remains to be confirmed whether our findings are broadly applicable. Nevertheless, we have identified several situations where mutants with altered sensitivity to the cognate QS signal are at a disadvantage when competing with the WT strain. Such conditions might have played roles in the evolution of LasR signal sensitivity in nature. We also find it interesting that not only did hyper-sensitivity decrease selectivity of LasR, but also hypo-sensitivity decreased selectivity. Perhaps, selectivity is tuned for life in complex polymicrobial habitats. This might also serve as a driver for the evolution of LasR sensitivity.

## MATERIALS AND METHODS

### Bacterial strains, growth conditions, and plasmids

Bacterial strains and plasmids are listed in Tables S1 and S2, respectively. Bacteria were grown in buffered lysogeny broth supplemented with 3-(*N*-morpholino)-propanesulfonic acid (LB-MOPS) (10 g tryptone, 5 g yeast extract, 5 g NaCl per liter with 50 mM 3-(*N*-morpholino)-propanesulfonic acid (MOPS), pH 7), casein broth containing 1% (wt/vol) sodium caseinate as the sole carbon and energy source (28, 40), casamino acids broth containing 1% (wt/vol) casamino acids as the sole carbon and energy source (40), or pyocyanin production medium (PPM; 20 g pancreatic digest of gelatin, 1.4 g magnesium chloride, 10 g potassium sulfate, 10 g glycerol per liter) (33), as indicated. For plasmid selection and maintenance, antibiotics were used at the following concentrations: *P. aeruginosa*, 30 µg/mL gentamicin (Gm); *Escherichia coli*, 10 µg/mL Gm. All chemicals and reagents were obtained from commercial sources. AHLs were dissolved in dimethyl sulfoxide (DMSO) and used at 1% of the final culture volume.

PAO1-LasR^hyper^ and -LasR^hypo^ are unmarked chromosomal mutants created in *P. aeruginosa* PAO1 using two-step allelic exchange. The allelic exchange vectors, pEXG2-lasR^A127L^ and pEXG2-lasR^L125F^, created in a previous study (6), were introduced to *P. aeruginosa* via conjugation with *E. coli* S17-1, and potential mutants were isolated as previously described (40). Mutations were confirmed by PCR amplification of *lasR* from culture lysates followed by Sanger sequencing. Strains used in competition experiments were marked with mCherry and gentamicin resistance at the neutral attTn7 site using the mini-Tn7 insertion system, without excision of the GmR marker (41).

### LasR sensitivity and selectivity measurements

LasR activity for signal selectivity experiments was measured in synthase-null *P. aeruginosa* (PAO-SC4) harboring pBBR-P*_rsaL_-gfp* using a previously developed method (5), with the modifications that AHL signals were dissolved in DMSO and used at 1% of the final culture volume, and the pBBR-P*_rsaL_*-gfp reporter was used rather than pPROBE-GT-P*_rsaL_*. Cultures were grown overnight, diluted 1:100 in LB-MOPS, and grown to mid-logarithmic phase before dilution to an optical density at 600 nm (OD_600_) of 0.01 in LB-MOPS. Cultures were then mixed with AHLs at the indicated concentrations and incubated for 16–18 hours in a 96-well-deep well plate, shaking at 37°C. LasR activity was measured as GFP fluorescence (excitation 490 nm, emission 520 nm) using a Synergy H1 microplate reader (Biotek Instruments) or a SpectraMax M3 microplate reader (Molecular Devices). Activity measurements were normalized by dividing by OD_600_ and subtracting background values of cultures incubated with a DMSO control. We note that the EC_50_ for 3OC12-HSL for WT PAO-SC4 was 2.7 µM, which is higher than in previous publications where EC_50_ has ranged from 600 to 800 nM (5, 6). Signals were purchased from a commercial source and used without further purification, and the higher 3OC12-HSL EC_50_ persisted across multiple batches of signal. In our study, LasR variant activity was measured in parallel experiments using the same stock of signals. As such, comparisons of relative activity between the variants are robust.

Selectivity scores were calculated using area under the curve calculated from dose response curves for each AHL up to a concentration of 100 µM using GraphPad Prism and a previously developed equation: selectivity score = AUC_3OC12-HSL_/ Σ (AUC_all other AHLs_) (5). Statistical analyses were conducted by using GraphPad Prism to perform one-way ANOVA with Dunnett’s multiple comparisons test, using WT as the control. We performed Shapiro-Wilk tests on our data sets to assess whether they have a normal (Gaussian) distribution. For all statistical analyses presented, the data pass the normality test (alpha = 0.05). Because pyocyanin production (Fig. 3E) and the competition with *B. multivorans* (Fig. 6) were normalized to WT within each independent experiment and WT was set to a value of 1, a Shapiro-Wilk test cannot be performed on these WT conditions. However, the underlying non-normalized data were assessed and passed the normality test.

### Transcriptional reporter assays

Plasmids were introduced to WT-PAO1 or LasR variants by electroporation and verified by PCR (42). Strains harboring pBBR-*gfp* with the indicated promoter regulating *gfp* expression were used to inoculate LB-MOPS supplemented with Gm. These cultures were grown overnight, then diluted 1:100 in LB-MOPS without antibiotic, and grown to mid-logarithmic phase. Cultures were diluted to an OD_600_ of 0.01 in LB-MOPS or casamino acids broth and dispensed in 300  µL volumes to the wells of a 48-well plate. Cultures were incubated shaking at 37°C, and GFP fluorescence and optical density (OD_600_) were measured every 15 minutes for 15 hours using a Synergy H1 microplate reader (BioTek Instruments) or Spark microplate reader (Tecan).

### Pyocyanin measurements

*P. aeruginosa* strains were grown overnight in LB-MOPS and then diluted 1:100 in PPM. Cultures were grown to mid-logarithmic phase, then diluted to an OD_600_ of 0.01 in PPM, and grown for 20–22 hours. Pyocyanin was extracted from 4 mL cultures with 2 mL chloroform. The organic layer containing pyocyanin was then mixed with 200 µL 0.1 N hydrochloric acid. A volume of 100 µL of the aqueous fraction was mixed with 900 µL water. Absorbance at 520 nm was used as a measure of pyocyanin concentration (33).

### *P. aeruginosa* growth and competition experiments

Because growth in casein broth requires proteases produced by *P. aeruginosa* cultures only at high cell densities, experiments in casein broth were initiated from stationary-phase cultures. To determine growth rates in casein broth or in casamino acids broth, initial cultures were grown overnight (22–24 hours) in LB-MOPS and then used to inoculate casein broth or casamino acids broth at an OD_600_ of 0.1 (28, 43). Growth was determined by plating serial dilutions of cultures at indicated time points on LB agar and counting colony-forming units.

For both casein broth and casamino acids broth coculture experiments, initial cultures were grown 22–24 hours in LB-MOPS, then diluted to a combined OD_600_ of 0.1 in the indicated medium. In each experiment, an unmarked “primary” strain was cocultured with a GmR “competitor” strain and inoculated at a starting frequency of 10%, 1%, or 0.1% GmR competitor. Cultures were incubated at 37°C, shaking at 250 rpm for 48 (casein broth) or 24 hours (casamino acids broth). Colony-forming units of GmR competitors and of the total population were determined by plating serial dilutions of the cultures on LB agar with or without 5 µg/mL Gm (28). Competitive indices were determined by dividing the end point GmR competitor frequency by the initial frequency. Statistical analyses were conducted by using GraphPad Prism to perform one-sample *t*-tests with a hypothetical value of 1 for competitive indices or one-way ANOVA with Dunnett’s multiple comparisons test for the significance of final competitor frequency.

### *Burkholderia multivorans* competition experiments

Cocultures of *P. aeruginosa* and *B. multivorans* were grown in LB-MOPS at 37°C by using a previously reported method (31). To inoculate cocultures, pure cultures of each strain were grown overnight in LB-MOPS, then diluted 1:100 in fresh LB-MOPS. Cultures were then grown to mid-logarithmic phase, diluted again in fresh LB-MOPS to an OD_600_ of 0.1, and grown to an OD_600_ of 1 before combining. The strains were combined at a ratio of 1:1 and grown for 24 hours before plating to enumerate CFU of each species. LB agar with trimethoprim (100 µg/mL) was used to select for *P. aeruginosa,* and LB agar with gentamicin (10 µg/mL) was used to select for *B. multivorans*.

## SUPPLEMENTAL MATERIAL

Supplemental figures jb.00249-23-s0001.pdf Fig. S1 to S8.

Table S1 jb.00249-23-s0002.docx Bacterial strains used in this study.

Table S2 jb.00249-23-s0003.docx Plasmids used in this study.

Table S3 jb.00249-23-s0004.docx Activity of LasR variants.

ASM does not own the copyrights to Supplemental Material that may be linked to, or accessed through, an article. The authors have granted ASM a non-exclusive, world-wide license to publish the Supplemental Material files. Please contact the corresponding author directly for reuse.
