# Supplementary material for: A balancing act: investigations on the impact of altered signal sensitivity in bacterial quorum sensing
Source: J Bacteriol. 2023 Nov 27;205(12):e00249-23. doi: 10.1128/jb.00249-23 (PMC10729764; doi:10.1128/jb.00249-23)
Supplement: Supplemental figures — Fig. S1 to S8. [file jb.00249-23-s0001.pdf]

Supplemental figures

A balancing act: investigations on the impact of altered signal sensitivity in bacterial quorum sensing

Samantha Wellington Miranda & E. Peter Greenberg

Department of Microbiology, University of Washington, Seattle, Washington 98195

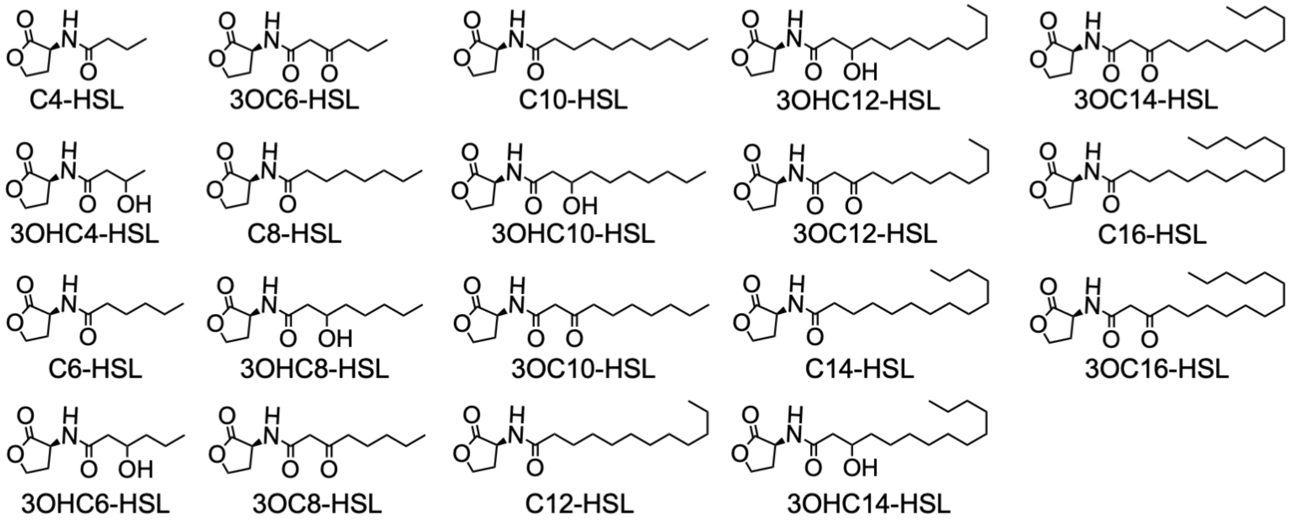

FIG S1 The structures and non-IUPAC names of acyl-homoserine lactone (AHL) signals used in this study. Several signals (C4-HSL, 3OHC4-HSL, C6-HSL, 3OC6-HSL, 3OHC6-HSL, C8-HSL, and 3OHC8-HSL) did not activate any LasR variant in this study.

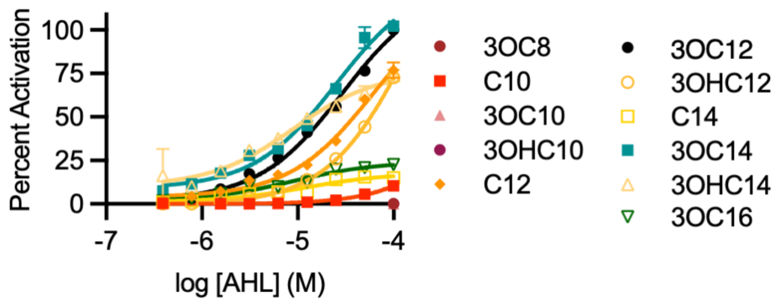

FIG S2 A hypo-sensitive LasR variant exhibits decreased selectivity. Activity of PAO-SC4-LasR^{R61L} in response to exogenous AHL signals, measured as GFP fluorescence from the transcriptional reporter pBBR-P$_{rsaL}$-*gfp*, normalized by optical density and to maximal activity stimulated by 3OC12-HSL.

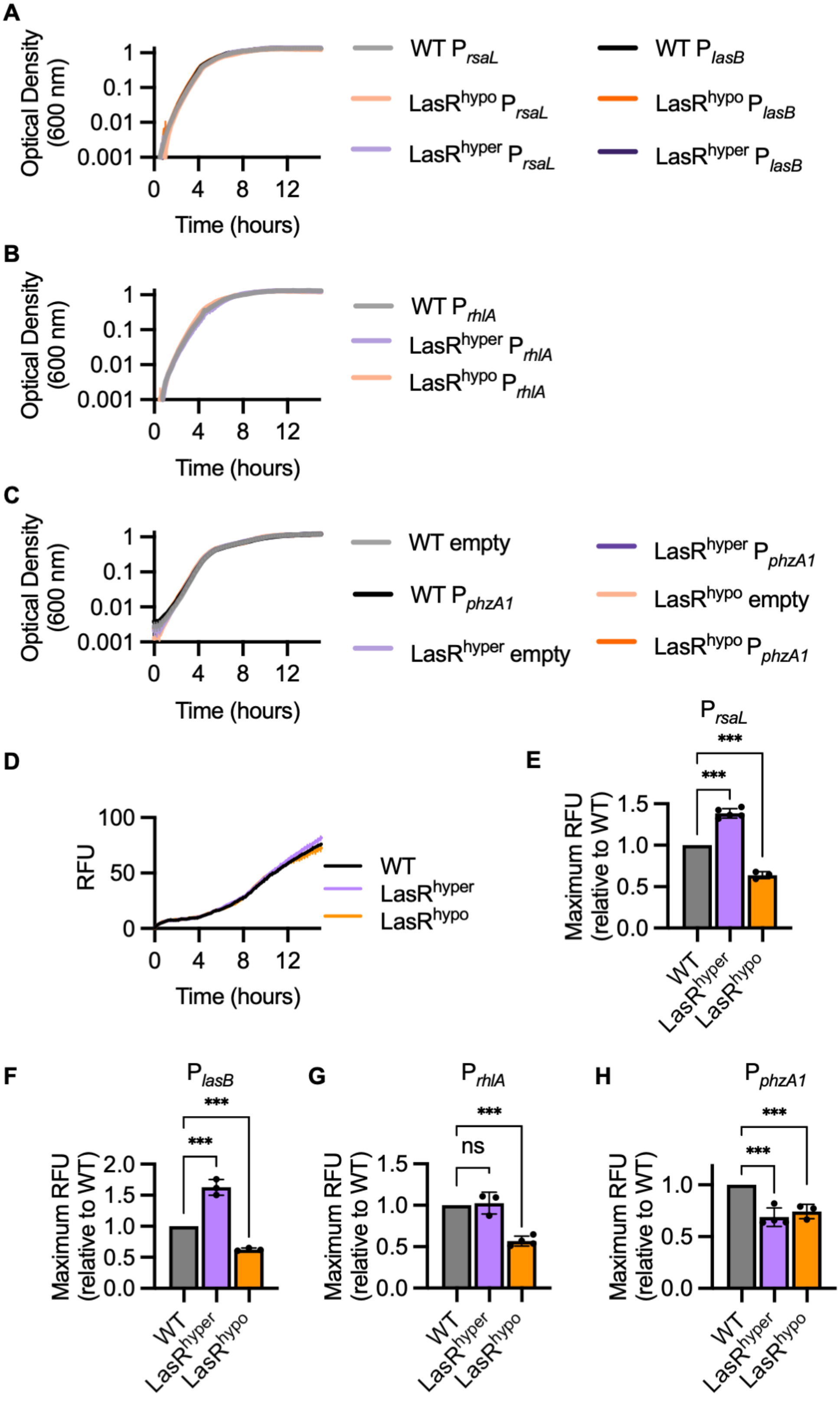

FIG S3 Growth of transcriptional reporter strains, signal from an empty vector control, and maximum reporter activity. (A-C) Growth of PAO1-WT, -LasR^{hyper}, or -LasR^{hypo} in LB-MOPS in 48-well plates measured as optical density at 600 nm. Strains harbored pBBR-*gfp* with the indicated promoter or the promoterless control "empty". (D) Relative Fluorescence Units (RFU) from a promoterless control, pBBR-*gfp*, in PAO1-WT and LasR variants. Data are mean and standard deviation of three biological replicates and are representative of n ≥ 3 independent experiments. (E-H) Maximum RFU measured from (E) pBBR-P_{rsaL}-*gfp* (F) -P_{lasB}-*gfp* (G) -P_{rhlA}-*gfp* or (H) -P_{phzA1}-*gfp* in the indicated strain over 15 h growth. Each data point is the mean of three biological replicates within an independent experiment normalized to the RFU from PAO1-WT harboring the same reporter, within the same independent experiment. Lines show the mean and standard deviation of the data points. Representative time course data is shown in Fig 3. *** indicates $P < 0.001$, ns is not significant (ANOVA).

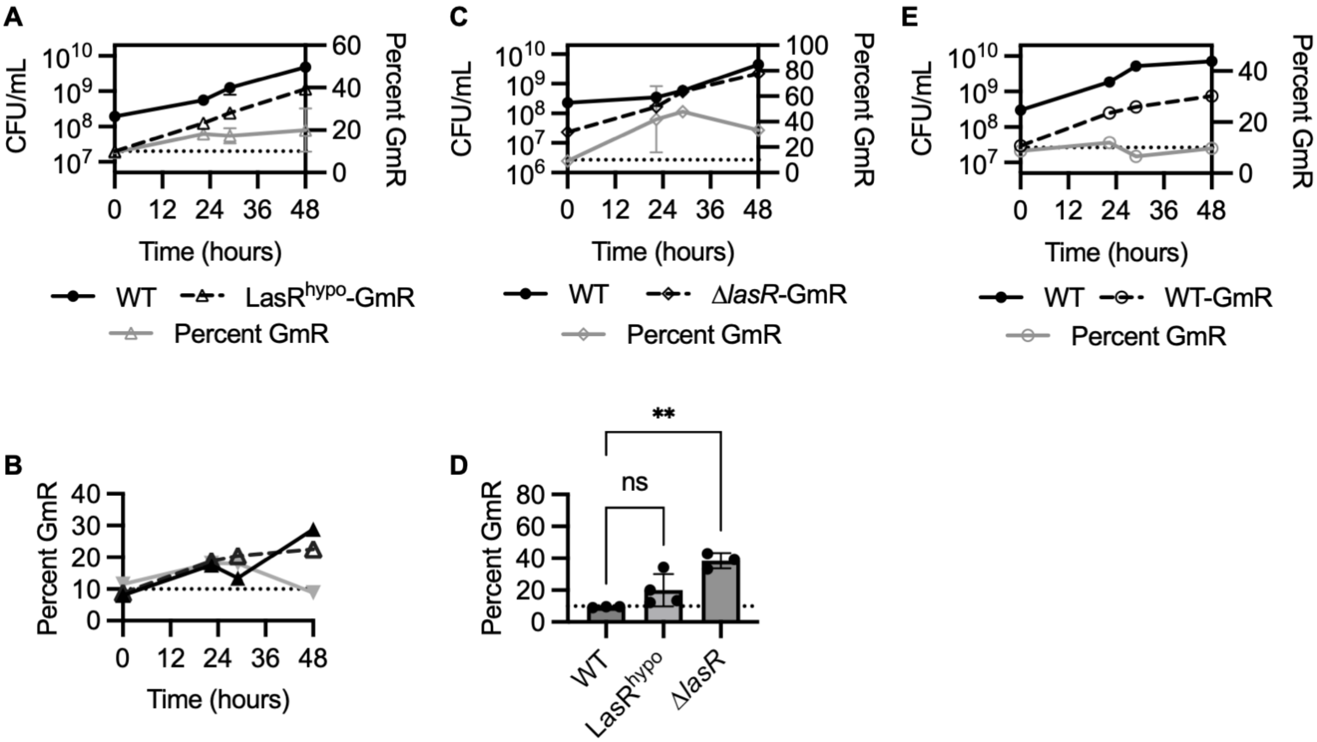

FIG S4 Growth of PAO1-WT and competitors in casein broth coculture. PAO1-WT and GmR competitors were inoculated in casein broth at a starting ratio of 9:1. The GmR competitor was (A) PAO1-LasR^{hypo}-GmR, (C) PAO1-Δ*lasR*-GmR or (E) PAO1-WT-GmR. Colony forming units (CFU) per mL of WT and competitor strains are shown on the left y-axis. Frequency of the GmR-competitor is indicated on the right y-axis. Data are the mean and standard error of three biological replicates and are representative of three independent experiments. (B) Frequency of PAO1-LasR^{hypo}-GmR in individual biological replicates from the competition shown in panel A. In two of three replicates, PAO1-LasR^{hypo}-GmR enriched, but in a third, although PAO1-LasR^{hypo}-GmR initially enriched, it ultimately returned to the starting frequency (indicated by the dotted line). Variability was observed across independent experiments of coculture between PAO1-WT and -LasR^{hypo}-GmR; one to three of three biological replicates enriched for PAO1-LasR^{hypo}-GmR, depending upon the experiment. (D) Frequency of GmR competitor after 48 hours coculture with PAO1-WT in casein broth. Each data point shows the mean of an independent experiment consisting of three biological replicates. Dashed line indicates GmR frequency of 10%. Lines show the mean and standard deviation of the data points. ** indicates $P < 0.01$, ns is not significant (ANOVA).

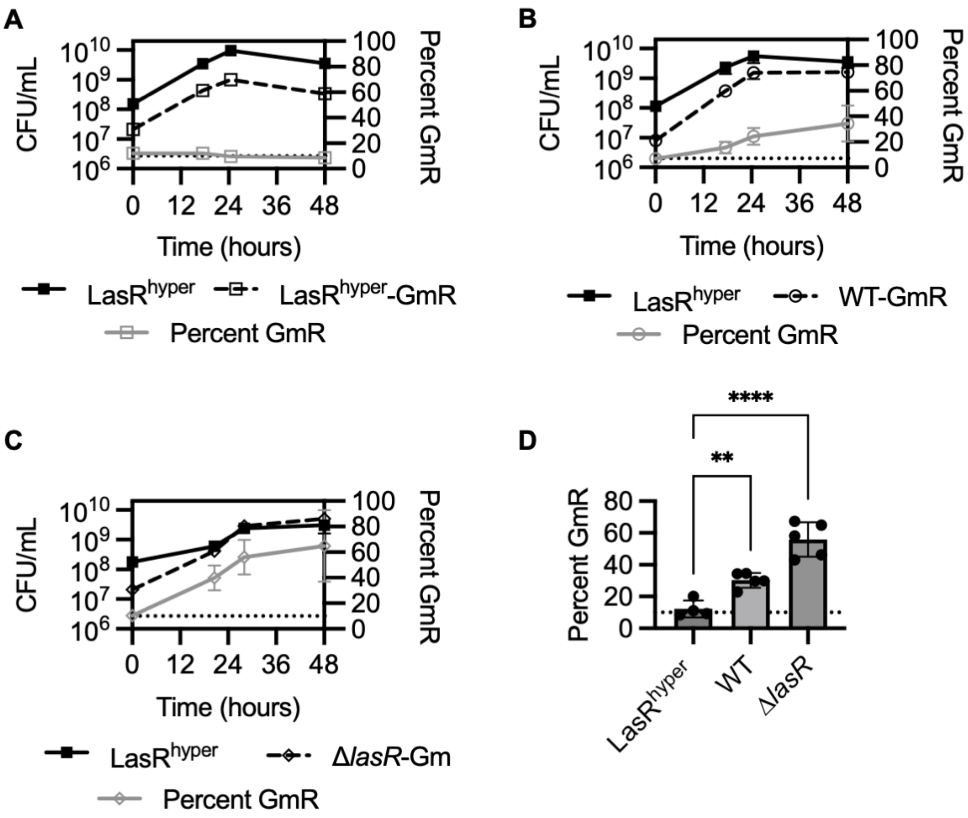

FIG S5 Growth of PAO1-LasR^{hyper} and competitors in casein broth coculture. PAO1-LasR^{hyper} and GmR competitors were inoculated in casein broth at a starting ratio of 9:1. The GmR competitor was (A) PAO1-LasR^{hyper}-GmR, (B) PAO1-WT-GmR, or (C) PAO1-Δ*lasR*-GmR. CFU/mL of PAO1-LasR^{hyper} and competitor strains are shown on the left y-axis. Frequency of the GmR competitor is indicated on the right y-axis. Data are the mean and standard error of three biological replicates and are representative of three independent experiments. (D) Frequency of GmR competitor after 48 hours coculture with PAO1-LasR^{hyper} in casein broth. Each data point shows the mean of an independent experiment consisting of three biological replicates. Lines show the mean and standard deviation of the data points. Dashed line indicates GmR frequency of 10%. **** indicates $P < 0.0001$, ** indicates $P < 0.01$ (ANOVA).

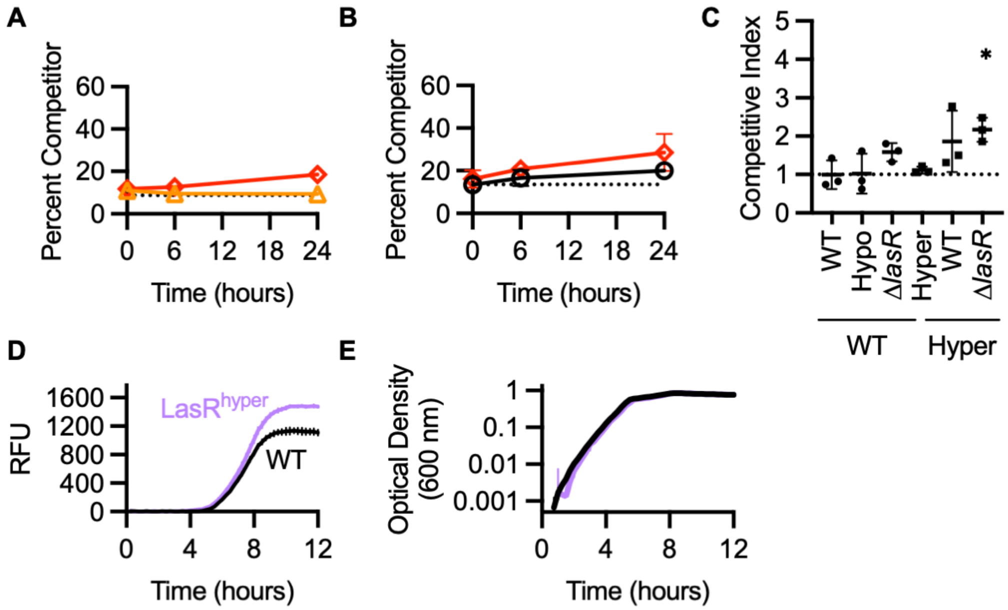

FIG S6 Fitness of LasR variants in casamino acids broth coculture. (A) Frequency of PAO1-LasR^{hypo}-GmR (triangles) or of PAO1-Δ*lasR*-GmR (diamonds) in casamino acids broth coculture with PAO1-WT. Data are the mean and standard deviation of three biological replicates. Dashed line indicates starting frequency. (B) Frequency of PAO1-WT-GmR (circles) or of PAO1-Δ*lasR*-GmR (diamonds) in casamino acids broth coculture with PAO1-LasR^{hyper}. Data are the mean and standard deviation of three biological replicates. (C) Competitive index after 22-24 hours of coculture in casamino acids broth. Cultures were inoculated with PAO1-WT (circles) or PAO1-LasR^{hyper} (squares) and the indicated GmR competitor at a ratio of 9:1 (primary culture: GmR competitor). Each data point shows the mean of an independent experiment consisting of three biological replicates. Lines show the mean and standard deviation of the data points. * indicates $P < 0.03$ (one-sample t-test, hypothetical mean = 1.0), all other comparisons were not significant. The dotted line marks a competitive index of 1, which indicates strains in the coculture were equally fit. (D) Transcriptional activation of the *rsaL* promoter measured as GFP fluorescence (RFU) from pBBR-P$_{rsaL}$-*gfp* in PAO1-WT (black) or -LasR^{hyper} (purple) grown in casamino acids broth. (E) Growth of PAO1-WT or -LasR^{hyper} harboring pBBR-P$_{rsaL}$-*gfp* in casamino acids broth measured as optical density at 600 nm. Data in D and E are the mean and standard deviation of three biological replicates and are representative of three independent experiments.

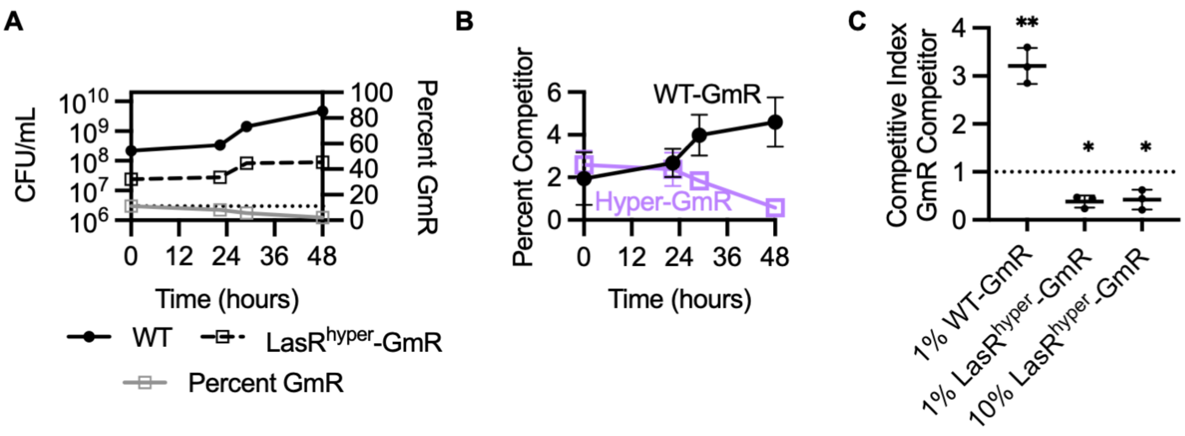

FIG S7 Impact of competitor frequency on PAO1-WT advantage over PAO1-LasR^{hyper}. (A) PAO1-WT and PAO1-LasR^{hyper}-GmR were inoculated in casein broth at a starting ratio of 9:1. CFU/mL of each strain is shown on the left y-axis. Frequency of PAO1-LasR^{hyper}-GmR is indicated on the right y-axis. Data are the mean and standard error of three biological replicates and are representative of three independent experiments. (B) Frequency of PAO1-WT-GmR (circles) in casein broth coculture with PAO1-LasR^{hyper} or of PAO1-LasR^{hyper}-GmR (squares) in casein broth coculture with PAO1-WT. Cultures were inoculated with a target frequency of 1% GmR competitor. Data are the mean and standard deviation of three biological replicates and are representative of three independent experiments. (C) Competitive index of the indicated GmR-competitor after 48 hours coculture in casein broth with PAO1-LasR^{hyper} for WT-GmR or with PAO1-WT for LasR^{hyper}-GmR. Cultures were inoculated with the indicated frequency of GmR competitor. Each data point shows the mean of an independent experiment consisting of three biological replicates. Lines show the mean and standard deviation of the data points. * indicates $P < 0.05$, ** indicates $P < 0.01$ (one-sample t-test, hypothetical mean = 1.0).

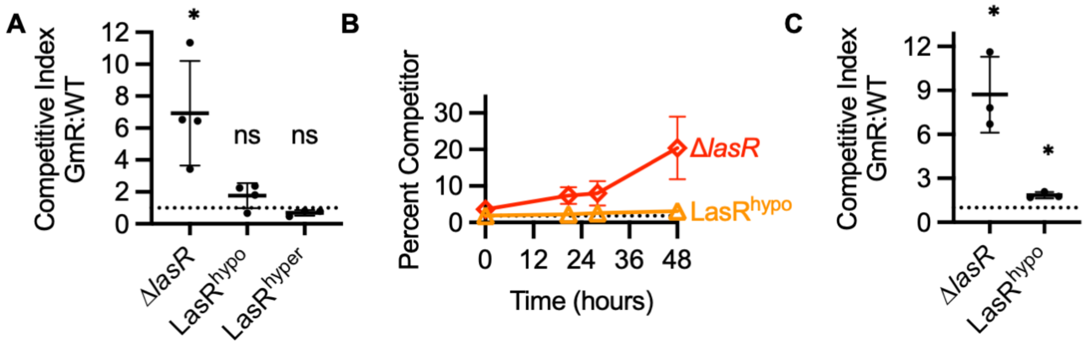

FIG S8 LasR variant invasion of PAO1-WT from a rare initial frequency. (A) Competitive index of the indicated GmR-competitor after 48 hours coculture in casein broth with PAO1-WT. Cultures were inoculated with 0.1% GmR competitor. Each data point shows the mean of an independent experiment consisting of three biological replicates. Lines show the mean and standard deviation of the data points. (B) Frequency of PAO1-Δ*lasR*-GmR (diamonds) or of PAO1-LasR^{hypo}-GmR (triangles) in casein broth coculture with PAO1-WT. Cultures were inoculated with a target frequency of 1% GmR competitor. Data are the mean and standard deviation of three biological replicates and are representative of three independent experiments. (C) Competitive index of the indicated GmR-competitor after 48 hours coculture in casein broth with PAO1-WT. Cultures were inoculated with 1% GmR competitor. Each data point shows the mean of an independent experiment consisting of three biological replicates. Lines show the mean and standard deviation of the data points. In A and C, * indicates $P < 0.05$, ns is not significant (one-sample t-test, hypothetical mean = 1.0).
