# Supplementary material for: A balancing act: investigations on the impact of altered signal sensitivity in bacterial quorum sensing
Source: J Bacteriol. 2023 Nov 27;205(12):e00249-23. doi: 10.1128/jb.00249-23 (PMC10729764; doi:10.1128/jb.00249-23)
Supplement: Table S1 — Bacterial strains used in this study. [file jb.00249-23-s0002.docx]

**Table S1** Bacterial strains used in this study

| Strain | Description | Source |
| --- | --- | --- |
| *Pseudomonas aeruginosa* | Strain PAO1, wild type (PAO1-WT) | (1) |
| PAO-SC4 | AHL synthase-null mutant; PAO1 with unmarked deletions of *lasI* and *rhII* | (2) |
| PAO-SC4-LasR^hyper^ | Unmarked chromosomal mutation in PAO-SC4 resulting in LasR A127L substitution | (3) |
| PAO-SC4-LasR^hypo^ | Unmarked chromosomal mutation in PAO-SC4 resulting in LasR L125F substitution | (3) |
| PAO-SC4-LasR^R61L^ | Unmarked chromosomal mutation in PAO-SC4 resulting in LasR R61L substitution | (3) |
| PAO1-LasR^hyper^ | Unmarked chromosomal mutation in PAO1 resulting in LasR A127L substitution | This study |
| PAO1-LasR^hypo^ | Unmarked chromosomal mutation in PAO1 resulting in LasR L125F substitution | This study |
| PAO1-∆*lasR* | Unmarked deletion of *lasR* in PAO1 | (4) |
| PAO1-WT-GmR | PAO1-WT with mCherry-GmR integrated into the neutral attTn7 site | (5) |
| PAO1-LasR^hyper^-GmR | PAO1-LasR^hyper^ with mCherry-GmR integrated into the attTn7 site | This study |
| PAO1-LasR^hypo^-GmR | PAO1-LasR^hypo^ with mCherry-GmR integrated into the attTn7 site | This study |
| PAO1-∆*lasR*-GmR | PAO1-∆*lasR* with mCherry-GmR integrated into the attTn7 site | (5) |
| *Burkholderia multivorans* | Strain AMT 0468-1 | (6) |
| *Escherichia coli* | Strain S17-1, used for conjugal transfer of plasmid DNA | (7) |

**REFERENCES**

1. Stover CK, Pham XQ, Erwin AL, Mizoguchi SD, Warrener P, Hickey MJ, Brinkman FSL, Hufnagle WO, Kowalik DJ, Lagrou M, Garber RL, Goltry L, Tolentino E, Westbrock-Wadman S, Yuan Y, Brody LL, Coulter SN, Folger KR, Kas A, Larbig K, Lim R, Smith K, Spencer D, Wong GKS, Wu Z, Paulsen IT, Reizer J, Saier MH, Hancock REW, Lory S, Olson MV. 2000. Complete genome sequence of Pseudomonas aeruginosa PAO1, an opportunistic pathogen. Nature 406:959-964. <https://doi.org/10.1038/35023079>

2. Wellington S, Greenberg EP. 2019. Quorum sensing signal selectivity and the potential for interspecies cross talk. mBio 10:e00146-19. <https://doi.org/10.1128/mbio.00146-19>

3. Wellington Miranda S, Cong Q, Schaefer AL, MacLeod EK, Zimenko A, Baker D, Greenberg EP. 2021. A covariation analysis reveals elements of selectivity in quorum sensing systems. eLife 10:e69169. <https://doi.org/10.7554/eLife.69169>

4. Wang M, Schaefer AL, Dandekar AA, Greenberg EP. 2015. Quorum sensing and policing of Pseudomonas aeruginosa social cheaters. Proc Natl Acad Sci U S A 112:2187-2191. <https://doi.org/10.1073/pnas.150070411>

5. Feng X, Kostylev M, Dandekar AA, Greenberg EP. 2019. Dynamics of cheater invasion in a cooperating population of *Pseudomonas aeruginosa*. Sci Rep 9:10190. <https://doi.org/10.1038/s41598-019-46651-5>

6. Smalley NE, An D, Parsek MR, Chandler JR, Dandekar AA. 2015. Quorum sensing protects *Pseudomonas aeruginosa* against cheating by other species in a laboratory coculture model. J Bacteriol 197:3154-3159. <https://doi.org/10.1128/jb.00482-15>

7. Simon R, Priefer U, Pühler A. 1983. A broad host range mobilization system for *in vivo* genetic engineering: Transposon mutagenesis in Gram negative bacteria. Bio/Technology 1:784-791. <https://doi.org/10.1038/nbt1183-784>
