# Supplementary material for: A balancing act: investigations on the impact of altered signal sensitivity in bacterial quorum sensing
Source: J Bacteriol. 2023 Nov 27;205(12):e00249-23. doi: 10.1128/jb.00249-23 (PMC10729764; doi:10.1128/jb.00249-23)
Supplement: Table S2 — Plasmids used in this study. [file jb.00249-23-s0003.docx]

**Table S2** Plasmids used in this study

| Plasmid | Description | Source |
| --- | --- | --- |
| pEXG2-lasR | Allelic exchange vector with pBR origin, *sacB*, GmR, and *lasR* coding sequencing along with 500 bp upstream and downstream *lasR* | (1) |
| pEXG2-lasR^A127L^ | pEXG2-lasR with site directed mutation to yield LasR^A127L^ (codon 127 GCG > CTG) | (1) |
| pEXG2-lasR^L125F^ | pEXG2-lasR with site directed mutation to yield LasR^L125F^ (codon 125 CTC > TTC) | (1) |
| pBBR-*gfp* | pBBR1-MCS5 with promoterless *gfp* transcriptional reporter, GmR | (2) |
| pBBR-P*_rsaL_-gfp* | pBBR-*gfp* with *gfp* under the control of the *rsaL* promoter (-82 to +29) | (3) |
| pBBR-P*_rhlA_-gfp* | pBBR-*gfp* with *gfp* under the control of the *rhlA* promoter (-502 to +31) | (4) |
| pBBR-P*_lasB_-gfp* | pBBR-*gfp* with *gfp* under the control of the *lasB* promoter (-336 to +3) | This study ^a^ |
| pBBR-P*_phzA1_-gfp* | pBBR-*gfp* with *gfp* under the control of the *phzA1* promoter (-395 to +79) | This study ^a^ |
| pUC18-mini-Tn7-Gm-mCherry | Suicide delivery vector for integration of mCherry and gentamicin resistance cassette in the attTn7 site | (5) |
| pTNS2 | Helper plasmid for integrating mCherry-GmR into the attTn7 site | (6) |

^a^ This plasmid was a gift from C Perez and AA Dandekar, generated using *E. coli*-mediated DNA assembly (7).

**REFERENCES**

1. Wellington Miranda S, Cong Q, Schaefer AL, MacLeod EK, Zimenko A, Baker D, Greenberg EP. 2021. A covariation analysis reveals elements of selectivity in quorum sensing systems. eLife 10:e69169. <https://doi.org/10.7554/eLife.69169>

2. Smalley NE, Schaefer AL, Asfahl KL, Perez C, Greenberg EP, Dandekar AA. 2022. Evolution of the quorum sensing regulon in cooperating populations of *Pseudomonas aeruginosa*. mBio 13:e00161-22. <https://doi.org/10.1128/mbio.00161-22>

3. Schwartzkopf CM, Robinson AJ, Ellenbecker M, Faith DR, Schmidt AK, Brooks DM, Lewerke L, Voronina E, Dandekar AA, Secor PR. 2023. Tripartite interactions between filamentous Pf4 bacteriophage, *Pseudomonas aeruginosa*, and bacterivorous nematodes. PLoS Pathog 19:e1010925. <https://doi.org/10.1371/journal.ppat.1010925>

4. Asfahl KL, Smalley NE, Chang AP, Dandekar AA. 2022. Genetic and transcriptomic characteristics of RhlR-dependent quorum sensing in cystic fibrosis isolates of *Pseudomonas aeruginosa*. mSystems 7:e00113-22. <https://doi.org/10.1128/msystems.00113-22>

5. Zhao K, Tseng BS, Beckerman B, Jin F, Gibiansky ML, Harrison JJ, Luijten E, Parsek MR, Wong GCL. 2013. Psl trails guide exploration and microcolony formation in *Pseudomonas aeruginosa* biofilms. Nature 497:388-391. <https://doi.org/10.1038/nature12155>

6. Choi K-H, Schweizer HP. 2006. Mini-Tn7 insertion in bacteria with single attTn7 sites: Example *Pseudomonas aeruginosa*. Nat Protoc 1:153-161. <https://doi.org/10.1038/nprot.2006.24>

7. Kostylev M, Otwell AE, Richardson RE, Suzuki Y. 2015. Cloning should be simple: *Escherichia coli* DH5α-mediated assembly of multiple DNA fragments with short end homologies. PLoS One 10:e0137466. <https://doi.org/10.1371/journal.pone.0137466>
