# Supplementary material for: A balancing act: investigations on the impact of altered signal sensitivity in bacterial quorum sensing
Source: J Bacteriol. 2023 Nov 27;205(12):e00249-23. doi: 10.1128/jb.00249-23 (PMC10729764; doi:10.1128/jb.00249-23)
Supplement: Table S3 — Activity of LasR variants. [file jb.00249-23-s0004.docx]

**Table S3 Activity of LasR variants measured in PAO-SC4 with pBBR-P*_rsaL_-gfp***

Values in the top row of each cell are EC_50_ ± standard error of the mean calculated at least from three independent experiments. “—” indicates EC_50_ was not resolved or > 50 μM. Values in the bottom row of each cell are relative LasR activity stimulated by 100 μM signal, normalized to maximum activity with 3OC12-HSL.

| Signal | WT | LasR^hyper^ | LasR^hypo^ | LasR^R61L^ |
| --- | --- | --- | --- | --- |
| 3OC8-HSL | — | 17.7 ± 2.0 μM | — | — |
|  | 8.4 ± 2.4% | 23.9 ± 4.4% | 5.5 ± 1.3% | < 1% |
| C10-HSL | — | — | — | — |
|  | 1.8 ± 0.3% | 3.2 ± 0.9% | 19.0 ± 7.5% | 11.1 ± 1.3% |
| 3OC10-HSL | — | 29.3 ± 12.2 μM | — | — |
|  | 17.2 ± 6.4% | 39.4 ± 7.8% | 62.0 ± 9.1% | < 1% |
| 3OHC10-HSL | — | — | — | — |
|  | 3.1 ± 2.5% | < 1 % | 18.1 ± 5.7% | < 1% |
| C12-HSL | 40.7 ± 7.4 μM | 11.8 ± 9.9 μM | — | 46.1 ± 21.1 μM |
|  | 22.0 ± 5.9% | 37.5 ± 7.9% | 23.0 ± 6.2% | 65.4 ± 9.8% |
| 3OC12-HSL | **2.7 ± 0.5 μM** | **0.42 ± 0.09 μM** | **27.9 ± 5.7 μM** | **> 50 μM** |
|  | **100%** | **100%** | **100%** | **100%** |
| 3OHC12-HSL | 49.6 ± 5.7 μM | 0.14 ± 0.01 μM | — | — |
|  | 48.1 ± 11.7% | 54.2 ± 12.1% | 47.0 ± 11.9% | 67.0 ± 4.2% |
| C14-HSL | 42.4 ± 22.3 μM | 10.2 ± 4.6 μM | 37.5 ± 11.9 μM | 9.6 ± 3.5 μM |
|  | 10.9 ± 2.7% | 24.6 ± 0.9% | 8.8 ± 3.4% | 20.1 ± 1.1% |
| 3OC14-HSL | 3.8 ± 0.5 μM | 0.24 ± 0.06 μM | 12.6 ± 2.0 μM | 18.6 ± 5.2 μM |
|  | 73.6 ± 10.4% | 104.2 ± 11.0% | 55.9 ± 9.6% | 110.9 ± 9.4% |
| 3OHC14-HSL | 4.9 ± 1.0 μM | 0.87 ± 0.15 μM | 12.4 ± 4.0 μM | 3.7 ± 0.3 μM |
|  | 31.9 ± 11.6% | 59.4 ± 6.9% | 23.6 ± 10.1% | 65.8 ± 11.5% |
| 3OC16-HSL | 15.4 ± 5.3 μM | 2.9 ± 1.1 μM | 8.3 ± 1.5 μM | 6.9 ± 0.8 μM |
|  | 30.9 ± 1.6% | 64.5 ± 10.7% | 22.4 ± 4.7% | 31.3 ± 4.0 % |
